# [Retracted] Inhibition of the transcription factor Sp1 suppresses colon cancer stem cell growth and induces apoptosis *in vitro* and in nude mouse xenografts

**DOI:** 10.3892/or.2026.9220

**Published:** 2026-11-09

**Authors:** Yingying Zhao, Wenjing Zhang, Zheng Guo, Feng Ma, Yao Wu, Yang Bai, Wei Gong, Ye Chen, Tianming Cheng, Fachao Zhi, Yali Zhang, Jide Wang, Bo Jiang

Oncol Rep 30: 1782-1792, 2013; DOI: 10.3892/or.2013.2627

Following the publication of this paper, it was drawn to the Editor's attention by a concerned reader that, regarding the immunohistochemical experiments shown in Fig. 1C on p. 1785, the fluorescence images labelled as ‘SW480 CSC’ and ‘HCT116 CSC’ (representing two different cell lines) appeared to show the same microscopic field after rotation, and with resizing of the images. In addition, the SW480 E-cadherin protein bands in Fig. 2D on p. 1786 appeared to be strikingly similar to the DLD1 and SW1116 Snail1 bands shown in Fig. 1A of an earlier published paper with the author Bo Jiang in common, which appeared in the journal *European Journal of Cancer*, where the image in Fig. 2D had been rotated by 180°, and in the two papers, these data were shown to represent different proteins, cell lines and experimental conditions.

Given that the abovementioned issues have come to light, the Editor of *Oncology Reports* has decided that this paper should be retracted from the Journal on account of a lack of confidence in the presented data. The authors were asked for an explanation to account for these concerns, but the Editorial Office did not receive a reply. The Editor apologizes to the readership for any inconvenience caused.

